# Intelligent risk prediction in public health using wearable device data

**DOI:** 10.1038/s41746-022-00701-x

**Published:** 2022-10-13

**Authors:** Marium M. Raza, Kaushik P. Venkatesh, Joseph C. Kvedar

**Affiliations:** Harvard Medical School, Boston, MA USA

**Keywords:** Preventive medicine, Medical research

## Abstract

The importance of infection risk prediction as a key public health measure has only been underscored by the COVID-19 pandemic. In a recent study, researchers use machine learning to develop an algorithm that predicts the risk of COVID-19 infection, by combining biometric data from wearable devices like Fitbit, with electronic symptom surveys. In doing so, they aim to increase the efficiency of test allocation when tracking disease spread in resource-limited settings. But the implications of technology that applies data from wearables stretch far beyond infection monitoring into healthcare delivery and research. The adoption and implementation of this type of technology will depend on regulation, impact on patient outcomes, and cost savings.

Wearable devices, such as smart watches, armbands, and glasses are rapidly becoming part of people’s daily lives. Many of these devices integrate biometric sensors and can be used to record and analyze health markers. The integration of these biometrics with other forms of patient data has broad implications, not only for healthcare interventions but also for the generation of data for health research in large populations. One potential application is within the infectious disease and public health. Combining wearables data with emergency room data, online search or social media^1^, health records, and survey results may allow for highly accurate infection risk prediction and timely intervention.

Shandhi et al.^2^ developed a model for risk prediction using digital biomarker data from wearables and symptom surveys to predict whether individuals are likely to be positive or negative for COVID-19 before they take a diagnostic test. The intelligent model was designed using machine learning to classify these potential positive and negative cases. The model aggregated data from the CovIdentify platform and the MyPHD study, both of which integrate commercial wearable device data and electronic symptom surveys. Biometrics measured included resting heart rate and step count. The model was validated separately within three cohorts: participants with both high-frequency and device-reported daily values, participants with high-frequency data only, and participants with high-frequency Fitbit data only.

Shandhi et al. argue that one application of their model is to allocate diagnostic testing resources more efficiently to measure disease spread, as compared to the conventional process of random allocation. The CovIdentify model ultimately did improve the positivity rate of COVID-19 diagnostic testing up to 6.5-fold when compared against random allocation, suggesting it could be successful if executed on a large scale. Additionally, by using the digital biomarker of resting heart rate as part of their model, Shandhi et al. demonstrate the potential of solely using physiological data from wearables to develop an intelligent model, which could be relevant in a resource-limited setting. Specifically, they found that differences in biometrics were significant between COVID-19 positive and negative groups as early as ten days prior to the diagnostic test date.

Limitations of this approach include potential bias in data generation. The participants in this study brought their own wearable devices, limiting eligibility to those who had access to wearable devices. Additionally, many different types of devices were used, and data from those devices had to be integrated with electronic symptom surveys. Without a standardized process for data fusion and processing, it would be difficult to scale up an intelligent model. Finally, symptoms were only most predictive shortly prior to participants' testing. This raises uncertainty about whether the time to testing is truly shortened and whether that decrease in time to testing is clinically meaningful.

Nevertheless, the improved positivity rate and patterns found before the diagnostic test date support the utility of wearable device data to identify more disease cases in less time, with fewer resources. Models like CovIdentify could also be used to notify infected individuals of hospital ER wait times via mobile devices and inform hospitals of real-time community infection patterns to optimize clinical workflow. Additionally, private organizations, in health care and beyond, may also be incentivized to adopt such technology to minimize workplace infections. With appropriate ethical measures, models like CovIdentify could be used to flag high-infection-risk workers and to inform contact tracing.

Intelligent risk prediction models also offer opportunities for public health beyond infection control, including targeted health promotion and treatment adherence. Future applications include using wearable and patient data to inform interventions for chronic conditions like heart disease^3^, diabetes, and obesity. For example, daily step count and heart rate data can be integrated with self-reported diet and weight to identify at-risk individuals as well as monitor lifestyle modification progress in real-time. Deidentified population-level data can also be used to monitor risk factors, identify at-risk populations^4^, and discern care gaps with new levels of precision.

Ultimately, the adoption and implementation of multimodal risk prediction models depend on their healthcare cost savings, impact on patient and population outcomes, and guidance from regulators. Providers may be motivated to adopt if the models decrease time to treatment and streamline diagnostic resource allocation, particularly within episodic and other value-based payment models. Where resultant cost savings outweigh the technology costs, providers could simply pay for risk prediction models as a cost of business. Where these models improve outcomes and decrease costs, payers^5^ may also want to reimburse the use of such technology through episodic/bundled payments, a New Technology Add-On Payment^6^, or another value-based payment model.

Local, state, and national governments can affect the utilization of this technology through direct implementation and broader regulation. In terms of implementation, given the potential of preventative medicine to decrease healthcare spending and decrease disease burden, risk prediction models could be subsidized and/or implemented by public health agencies. For example, models like CovIdentify could seamlessly be implemented through existing exposure notification software like New York City’s COVID Alert NY^7^ smartphone app. At a national level, the FDA must create clear guidelines as to which types of risk prediction lie in their purview, perhaps building on new guidance in the FDA Digital Health Software Precertification (Pre-Cert) Program^8^ to standardize the AI technology approval process.

Altogether, the CovIdentify model offers an example of risk prediction using wearable and survey data to inform diagnostic testing allocations. More proximally, the CovIdentify model can be used to inform diagnostics allocation for infectious diseases, implementable at the individual, organizational, and population levels—with particular benefits to under-resourced settings. Multimodal AI-driven risk prediction more broadly has implications across health care, for diagnostics, prognostics, and clinical decision-making. How—and whether—these risk prediction innovations like CovIdentify are adopted and implemented depends on methods of regulatory approval as well as public and private reimbursement and/or subsidy. Nevertheless, intelligent allocation and risk prediction innovations have an important part to play in the future of personalized medicine and precision public health.
